# Unmasking the Parasite: When Giardiasis Looks Like Gastroenteritis

**DOI:** 10.7759/cureus.106370

**Published:** 2026-04-03

**Authors:** Abeer Qasim, Rayan Alataa, Sameer Kandhi, Balakrishnan Arivalagan, Harish Patel

**Affiliations:** 1 Gastroenterology, BronxCare Health System, New York, USA; 2 Internal Medicine, BronxCare Health System, New York, USA; 3 Gastroenterology and Hepatology, BronxCare Health System, New York, USA

**Keywords:** endoscopic biopsy, eosinophilia, eosinophilic duodenitis, giardiasis, malabsorption

## Abstract

Giardiasis is a protozoal infection that typically presents with diarrhea, abdominal pain, and malabsorption. Peripheral eosinophilia is unusual and may confound diagnosis by suggesting eosinophilic gastrointestinal disease (EGID). We present a 41-year-old woman with abdominal pain, diarrhea, malabsorption, and peripheral eosinophilia. Endoscopic biopsy performed shortly after anti-parasitic therapy demonstrated eosinophilic duodenitis, raising concern for eosinophilic gastroenteritis. However, repeat biopsy after recovery revealed resolution of eosinophilia, excluding EGID. This case highlights the importance of timing diagnostic endoscopy after giardiasis treatment to avoid misdiagnosis.

## Introduction

*Giardia duodenalis* is a flagellated protozoan that causes giardiasis, one of the most common enteric parasitic infections worldwide [1]. Globally, giardiasis affects approximately 200 million people annually, with a disproportionately high prevalence in developing countries where access to clean water and sanitation is limited. In the United States, an estimated 1.2 million cases occur each year, though the true burden is likely underestimated due to asymptomatic carriage and under-reporting [2]. Locally acquired infections are frequently missed due to the widespread misconception that giardiasis is exclusively a travel-associated illness, leading to inadequate testing in patients without a relevant travel history.

The infection is transmitted via the ingestion of cysts, the environmentally resistant form of the parasite; typically through contaminated water, food, or direct person-to-person contact [3]. Risk factors include exposure to recreational water, contact with infected animals, and poor hand hygiene. The clinical presentation of giardiasis is highly variable. While many individuals remain asymptomatic, symptomatic patients typically present with abdominal pain, nausea, bloating, flatulence, watery diarrhea, and steatorrhea.

Diagnostic challenges arise when giardiasis presents with atypical features, such as peripheral eosinophilia, which can mimic eosinophilic gastroenteritis (EGE), a subtype of eosinophilic gastrointestinal diseases (EGIDs). Both conditions can present with chronic abdominal pain, diarrhea, malabsorption, and peripheral eosinophilia, making them clinically indistinguishable without careful histologic and temporal correlation. EGE is a distinct immune-mediated disorder that typically requires systemic steroid therapy, whereas giardiasis is treated with targeted antimicrobial agents such as metronidazole or tinidazole [4].

We present the case of a 41-year-old female who initially presented with epigastric pain, iron deficiency anemia (IDA), and peripheral eosinophilia. She underwent an upper endoscopy (EGD) with findings highly suggestive of eosinophilic gastroenteritis. However, it was later discovered that she had recently been treated with metronidazole for giardiasis. A repeat endoscopic evaluation demonstrated significant improvement in mucosal eosinophilic infiltration. This case underscores the critical importance of appropriately timing endoscopic evaluation after the treatment of parasitic infections to accurately diagnose suspected EGIDs, particularly given the lack of standardized diagnostic guidelines for these conditions.

## Case presentation

**Figure 1:**
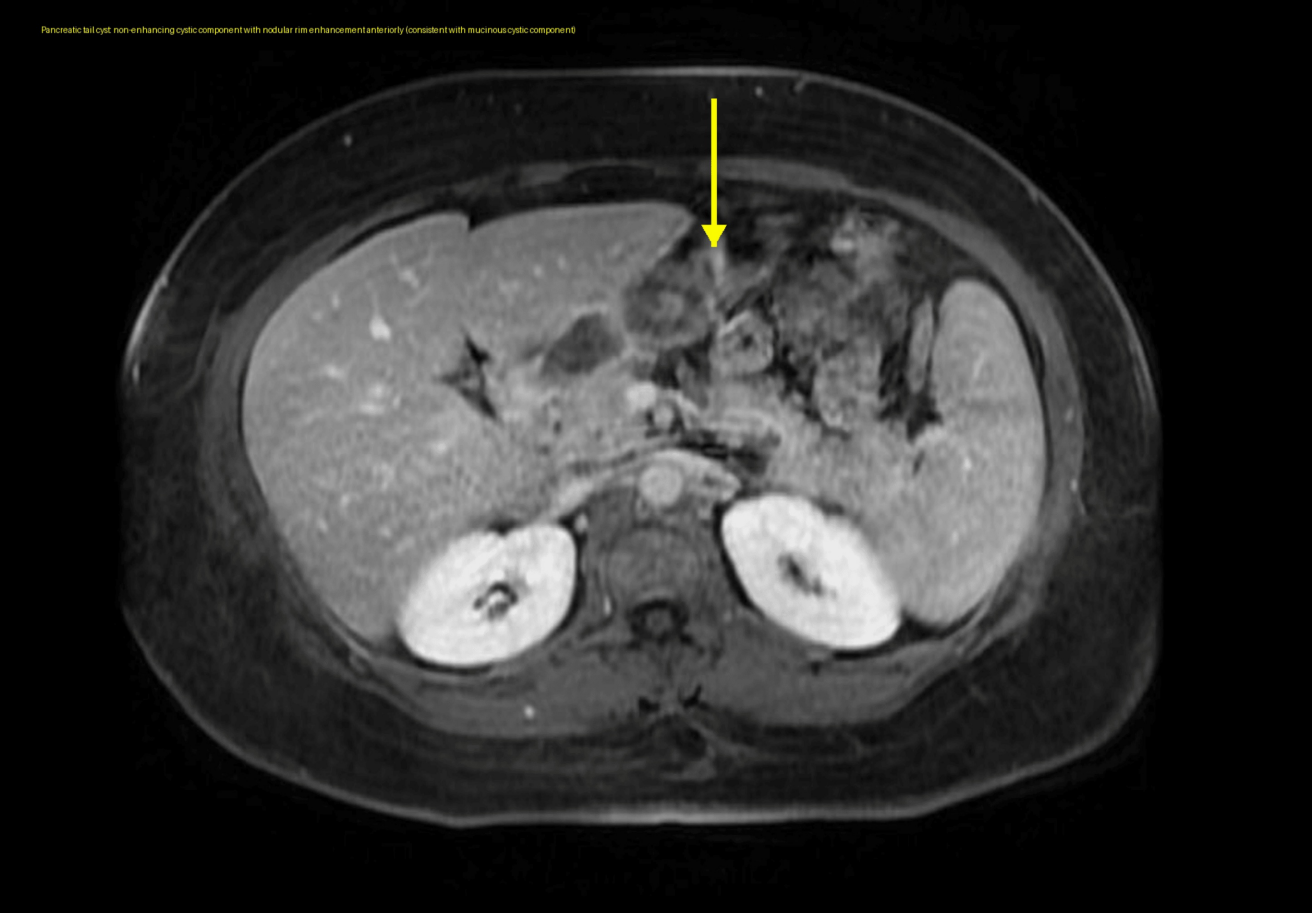
Axial post-contrast MRI demonstrating a pancreatic tail cyst with a non-enhancing cystic component and nodular rim enhancement anteriorly, consistent with a mucinous cystic component

**Figure 2:**
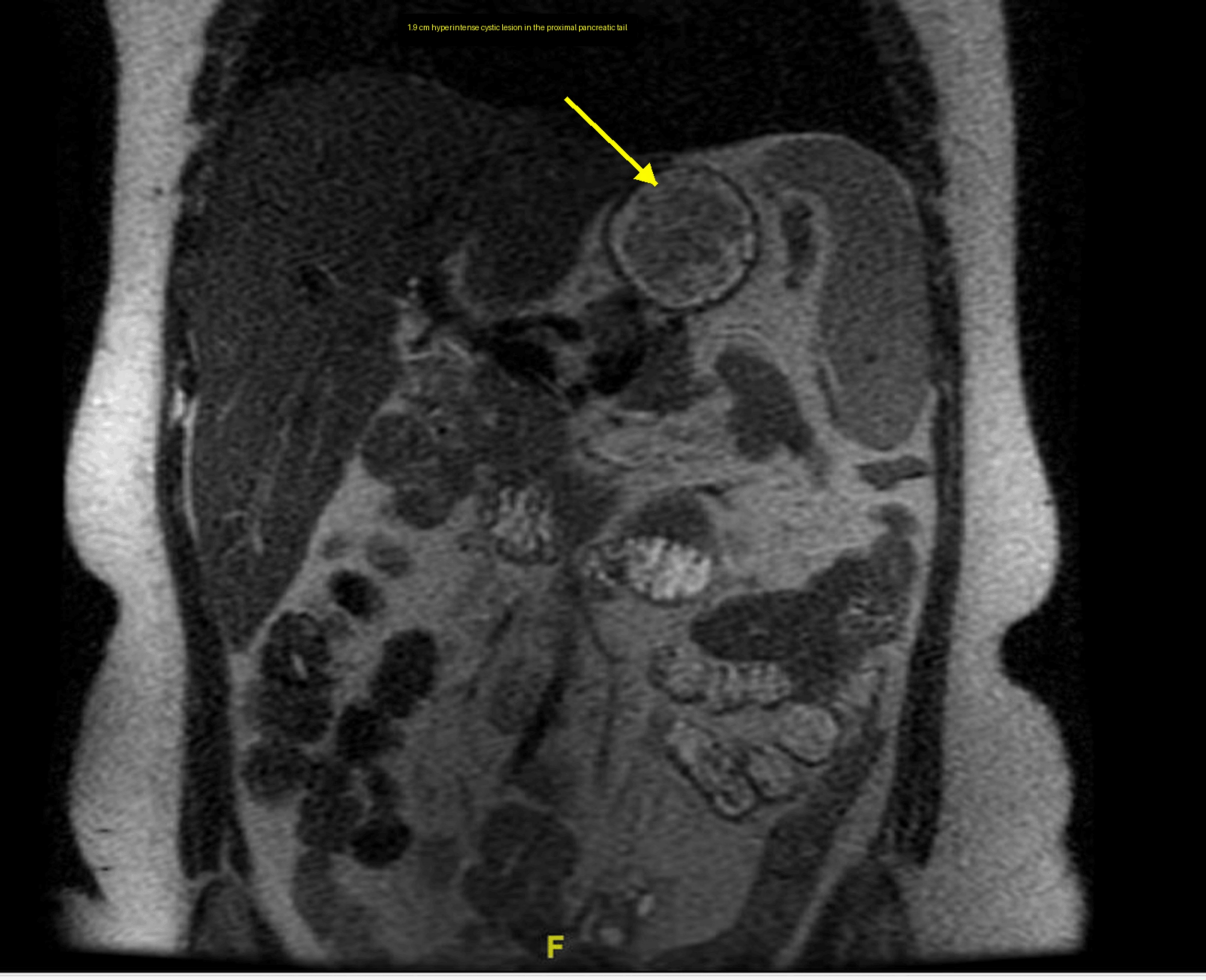
Coronal T2-weighted MRI showing a 1.9 cm hyperintense cystic lesion in the proximal pancreatic tail

This was a 41-year-old female with a past medical history of lactose intolerance and asthma (on inhaler treatment with albuterol and formoterol) who presented to the hospital for epigastric pain. The epigastric pain was chronic with an intermittent course for the last five months and a few acute flares, prompting her to have multiple emergency room (ER) visits in the last one to two months. The pain was sharp in nature and radiated to the left upper back. She also described having watery diarrhea over the past several weeks. Labs drawn upon presentation showed severe microcytic hypochromic anemia; however, there was no overt gastrointestinal/genitourinary blood loss clinically. A computed tomography (CT) scan of the abdomen and pelvis with intravenous contrast revealed a 1.9 x 2 cm right peritoneal fluid collection and a hypodense lesion in the pancreatic tail. Subsequently, magnetic resonance imaging (MRI) of the abdomen with magnetic resonance cholangiopancreatography (MRCP) was performed. This imaging demonstrated a pancreatic cystic lesion with a nodular component, raising concern for a mucinous cystic neoplasm, though no biliary ductal dilatation was observed (Figures 1-2). 

Further laboratory workup (summarized in Table 1) demonstrated peripheral eosinophilia (absolute eosinophil count: 800 cells/uL) and iron deficiency anemia. Biochemical evidence of malabsorption was present, including low serum calcium, total protein, albumin, and vitamin D levels, while serum folate and vitamin B12 levels remained normal. An immunological workup revealed decreased serum immunoglobulin levels, specifically low IgG, IgE, and IgM. Furthermore, her tissue transglutaminase antibody (celiac panel) was negative, and CA 19-9 was also negative.

**Table 1:** Laboratory findings MCV: Mean Corpuscular Volume; IgG/IgE/IgM/IgA: Immunoglobulin G/E/M/A; CA 19-9: Carbohydrate Antigen 19-9; HIV: Human Immunodeficiency Virus

Test	Result	Reference Range
Hemoglobin	11 g/dL	12.0-16.0 g/dL (F)
MCV (Mean Corpuscular Volume)	75 fL	80-100 fL
Absolute Eosinophil Count	800 cells/μL	0-500 cells/μL
Serum Iron	33 μg/dL	50-170 μg/dL
Ferritin	8 ng/mL	10-120 ng/mL
Serum Calcium	7 mg/dL	8.5-10.5 mg/dL
Total Protein	5 g/dL	6.0-8.3 g/dL
Serum Albumin	2.1 g/dL	3.5-5.5 g/dL
Vitamin D (25-OH)	19 ng/mL	30-100 ng/mL
Folate	5 ng/mL	>3.0 ng/mL
Vitamin B12	210 pg/mL	200-900 pg/mL
IgG	136 mg/dL	700-1600 mg/dL
IgE	15 IU/mL	<100 IU/mL
IgM	30 mg/dL	40-230 mg/dL
IgA	15 mg/dl	70–400 mg/dL
CA 19-9	10 U/mL	<37 U/mL
HIV Antibody	Negative	Negative

Because she was also being treated for pneumonia during that admission, with intravenous antibiotics (ceftriaxone and azithromycin), invasive evaluation was deferred until clinically optimized, with planning for upper endoscopy (EGD) along with endoscopic ultrasound (EUS) for a pancreatic cyst as an outpatient on an elective basis.

Her initial EGD, performed in June 2025, revealed multiple duodenal lymphoid follicles and a prominent papilla in the second portion of the duodenum, with no gross mucosal lesions in the stomach or esophagus. The planned EUS was deferred due to respiratory destabilization related to her asthma at the conclusion of the EGD. Mucosal biopsies obtained from the duodenum during this initial EGD demonstrated an infiltration of 50 eosinophils per high-power field (hpf) in the lamina propria, a finding consistent with eosinophilic duodenitis. Biopsies from the stomach and esophagus were unremarkable. Follow-up serological testing for strongyloidiasis was negative.

The patient subsequently underwent a repeat EGD and EUS in July 2025. The EUS confirmed multiple cystic lesions in the pancreatic tail, the largest measuring approximately 1 cm with a nodular component, alongside a small peripancreatic lymph node and a normal main pancreatic duct. Cyst fluid analysis was consistent with a mucinous cystic neoplasm (carcinoembryonic antigen (CEA) > 192 ng/mL, amylase 45 U/L). Additional duodenal biopsies taken during this second endoscopic evaluation revealed a marked improvement in eosinophilic infiltration, decreasing to 10-15 cells/hpf. Given the EUS findings, she was referred to the hepatobiliary clinic, where a follow-up MRI of the pancreas in six months was recommended.

The patient was again hospitalized later that month (July 2025) for abdominal pain and watery diarrhea. Given the discrepancy in her duodenal biopsy results across the two prior endoscopies and her persistent iron deficiency anemia, the gastroenterology team elected to perform a third EGD with multiple mucosal biopsies, along with a colonoscopy to evaluate for colonic involvement. A thorough chart review during that admission revealed that the patient had a stool study positive for *Giardia* in May 2025, for which she was treated with oral metronidazole (500 mg three times daily) for 5 days. This recent parasitic infection and its subsequent treatment were identified as a major confounder for the marked tissue eosinophilia observed during the initial June 2025 endoscopy. The repeat endoscopy during hospitalization no longer demonstrated duodenal mucosal eosinophilic infiltration. Pathology was also not suggestive of celiac disease. Colonoscopy was also unremarkable, with colonic mucosal biopsies showing no eosinophilic or lymphocytic infiltration.

During this admission, her infectious stool studies returned positive for *Clostridioides difficile *(*C. difficile*) toxin assay, for which she was started on oral vancomycin for *C. difficile* colitis, resulting in significant improvement in her diarrhea. Her epigastric pain also subsided, and she was discharged in stable condition with scheduled outpatient follow-up. Her epigastric pain also improved, and she was discharged in stable condition with follow-up.

**Figure 3:**
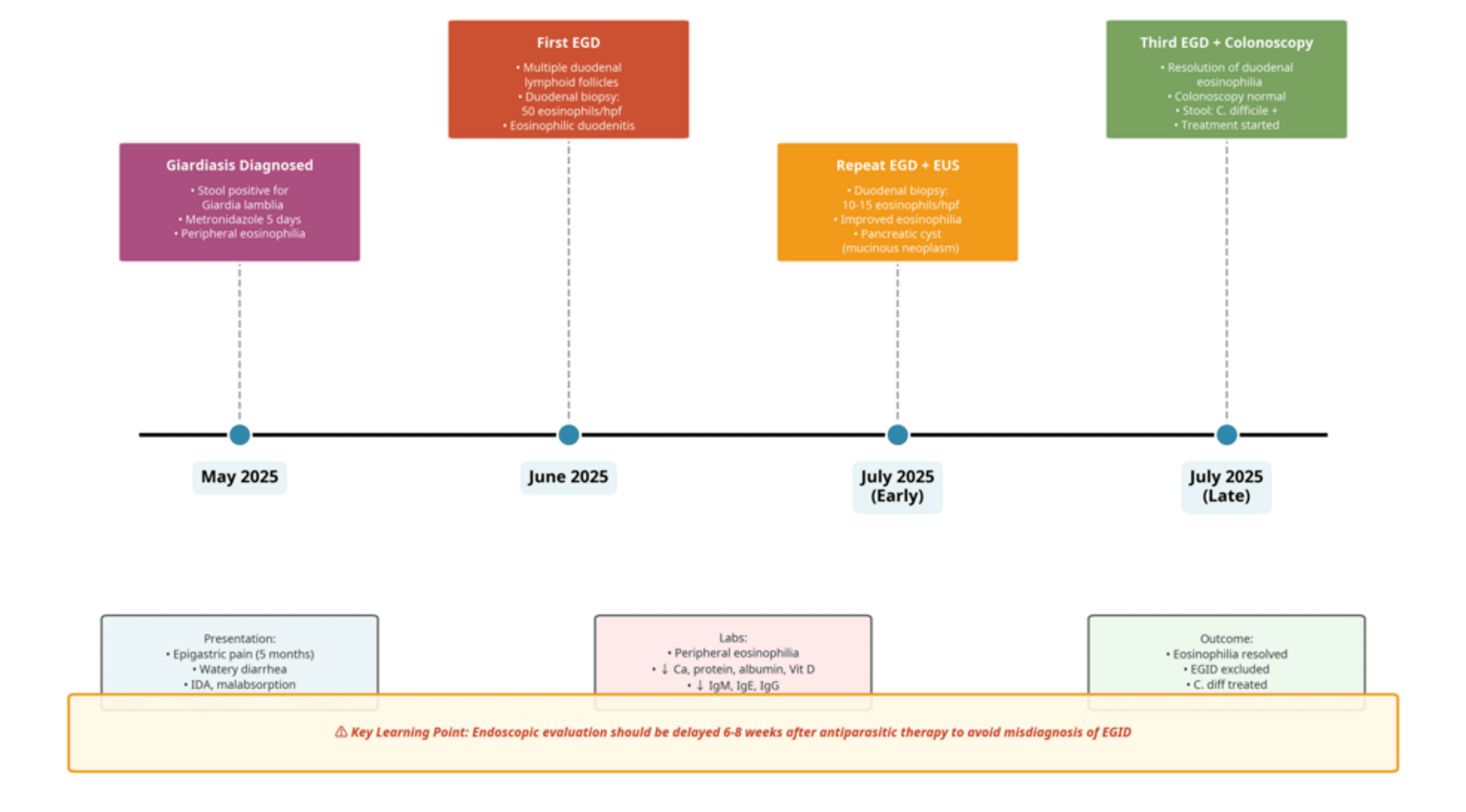
Clinical timeline of patient course

This complex clinical course (summarized in Figure 3) underscores the critical importance of a thorough historical review, specifically regarding recent parasitic infections and their treatment, when evaluating patients for suspected EGIDs. Furthermore, it highlights the necessity of performing a complete evaluation, including colonoscopy with biopsies, to exclude colonic involvement before considering systemic steroid therapy for presumed eosinophilic gastroenteritis.

## Discussion

Giardiasis, a common protozoal infection of the small intestine caused by *Giardia duodenalis* (also known as *G. intestinalis *or *G. lamblia*), presents with a wide clinical spectrum. While many individuals remain asymptomatic, symptomatic cases typically develop one to two weeks after infection and are characterized by watery diarrhea, bloating, flatulence, and abdominal discomfort [4,5]. In the United States, an estimated 1.2 million cases occur annually, although many remain undetected due to asymptomatic carriage. In 2012, the CDC documented 15,223 confirmed cases, with the highest incidence in children under 5 years of age and a regional concentration in northwestern states. Notably, Yoder et al. suggested that regional variations in incidence may partly reflect differences in reporting systems rather than true epidemiologic disparities [6].

The clinical presentation of giardiasis is variable. Common symptoms include abdominal discomfort, nausea, bloating, flatulence, and the passage of large, watery, greasy, and foul-smelling stools. In children, abdominal pain may occur with minimal or no diarrhea. Frequent loose stools can result in dehydration, and although less common, fever may also be present [7]. In acute infection, diarrhea is the predominant symptom, occurring in up to 90% of patients, while 70-75% also report abdominal cramps, bloating, and excessive gas. Chronic giardiasis may manifest as prolonged diarrhea, fatigue, weight loss, anorexia, and decreased appetite. Lactase deficiency often develops following infection and contributes to persistent gastrointestinal symptoms [8]. In our case, the patient initially presented with epigastric pain and subsequently developed diarrhea.

Giardiasis typically does not produce hematologic abnormalities, and peripheral eosinophilia is rarely observed. When present, eosinophilia can complicate the diagnostic process by raising suspicion for other conditions such as eosinophilic gastrointestinal diseases [9,10]. Only a limited number of cases have described eosinophilia associated with giardiasis [11]. A Brazilian study by Dos Santos and Vituri proposed that *Giardia*-derived allergens may trigger an eosinophilic response in susceptible individuals [12].

Malabsorption in giardiasis results from both direct mucosal injury by the parasite and the host’s inflammatory response, which disrupts intestinal absorptive and barrier functions. This process contributes to diarrhea, abdominal pain, anorexia, and weight loss, and may result in deficiencies of multiple micronutrients, including vitamin A, thiamine, folic acid, vitamin B12, and iron, ultimately leading to anemia [13]. In our patient, laboratory evaluation revealed iron deficiency anemia, hypocalcemia, hypoproteinemia, vitamin D deficiency, low total immunoglobulins, and elevated phosphorus, with preserved serum folate and vitamin B12 levels.

Giardiasis can be diagnosed using stool microscopy, stool antigen assays, or nucleic acid amplification tests (NAATs). Microscopy has limited sensitivity due to the intermittent shedding of cysts; however, diagnostic yield improves when three stool samples are collected on different days. Antigen detection assays and NAATs are faster, more sensitive, and more specific, making them valuable tools in high-incidence settings. Nonetheless, they should be viewed as complementary to microscopy rather than complete replacements [14].

Diagnosis of EGE, a subtype of EGIDs, requires the presence of gastrointestinal symptoms in conjunction with histologic evidence of pathologic tissue eosinophilia. Recommended thresholds include ≥30 eosinophils per hpf in the stomach, ≥52 eosinophils/hpf in the duodenum, and ≥56 eosinophils/hpf in the ileum [15]. Secondary causes must be excluded through stool and serum assays for parasitic infections, as well as evaluation for inflammatory bowel disease and neoplasia. Peripheral eosinophilia is seen in up to 70% of patients, and approximately two-thirds demonstrate elevated serum IgE levels. Malabsorptive features are also common, including steatorrhea, protein-losing enteropathy (manifested by hypoalbuminemia and hypoproteinemia), weight loss, failure to thrive, and micronutrient deficiencies such as iron, folate, and vitamin B12 [15]. 

In our case, the patient’s initial EGD, performed for the evaluation of abdominal pain and iron deficiency anemia, revealed marked duodenal eosinophilic infiltration of up to 50 cells per hpf. These findings, together with transient laboratory abnormalities, including peripheral eosinophilia and biochemical evidence of malabsorption, initially raised a strong suspicion for eosinophilic gastroenteritis. However, a repeat upper endoscopy performed four weeks later demonstrated complete resolution of mucosal eosinophilia without any therapy, leading us to re-examine her history. This review revealed a recently treated parasitic infection, giardiasis, approximately two months earlier, which likely accounted for the transient eosinophilic changes.

Based on our experience, we recommend deferring endoscopic evaluation for suspected EGIDs until at least six to eight weeks after completion of antiparasitic therapy. Procedures performed earlier may yield misleading eosinophilic findings, potentially resulting in a misdiagnosis of EGE and the inappropriate initiation of systemic corticosteroid therapy. While our recommendation is based on a single complex case, it aligns with the pathophysiological understanding of post-infectious mucosal healing. Further prospective studies are needed to establish and standardize the optimal interval for post-infectious endoscopic assessment.

## Conclusions

This case highlights critical diagnostic considerations when evaluating eosinophilic gastrointestinal diseases (EGIDs). Giardiasis can rarely present with peripheral eosinophilia and marked tissue eosinophilic infiltration, closely mimicking eosinophilic gastroenteritis. The resolution of eosinophilia following antiparasitic therapy in our patient supports a causal relationship between giardiasis and these atypical manifestations. The timing of diagnostic endoscopy is crucial. Endoscopic evaluation performed shortly after parasitic treatment may reveal transient eosinophilic infiltration that does not represent true EGID. We recommend deferring EGD with biopsy for at least six to eight weeks after the completion of antiparasitic therapy to avoid misdiagnosis.

Physicians should exclude secondary causes before diagnosing EGID through a comprehensive evaluation, including colonoscopy, a detailed history of prior parasitic infections, and stool ova and parasite testing. This approach is essential given the current paucity of standardized diagnostic guidelines for EGIDs and ensures appropriate management while avoiding unnecessary immunosuppressive therapy.
